# Some highlights of research on aging with invertebrates, 2010

**DOI:** 10.1111/j.1474-9726.2010.00649.x

**Published:** 2011-02

**Authors:** Linda Partridge

**Affiliations:** 1 Max Planck Institute for Biology of AgeingGleueler Strasse 55a, 50931 Köln, Germany; 2 Institute for Healthy Ageing, UCLDarwin Buidling, Gower St, London WC1E 6BT, UK

## Abstract

This annual review focuses on invertebrate model organisms, which continue to yield fundamental new insights into mechanisms of aging. This year, the budding yeast has been used to understand how asymmetrical partitioning of cellular constituents at cell division can produce a rejuvenated offspring from an aging parent. Blocking of sensation of carbon dioxide is shown to extend fly lifespan and to mediate the lifespan-shortening effect of sensory exposure to fermenting yeast. A new study of *daf-16*, the key forkhead transcription factor that mediates extension of lifespan by mutants in the insulin-signalling pathway in *Caenorhabditis elegans*, demonstrates that expression of tissue-specific isoforms with different patterns of response to upstream signalling mediates the highly pleiotropic effects of the pathway on lifespan and other traits. A new approach to manipulating mitochondrial activity in *Drosophila*, by introducing the yeast NADH-ubiquinone oxidoreductase, shows promise for understanding the role of mitochondrial reactive oxygen species in aging. An exciting new study of yeast and mammalian cells implicates deterioration of the nuclear pore, and consequent leakage of cytoplasmic components into the nucleus, as an important cause of aging in postmitotic tissues. Loss of, or damage to, chromosome-associated histones is also implicated in the determination of lifespan in yeast, worms and fruit flies. The relationship between functional aging, susceptibility to aging-related disease and lifespan itself are explored in two studies in *C. elegans*, the first examining the role of dietary restriction and reduced insulin signalling in cognitive decline and the second profiling aggregation of the proteome during aging. The invertebrates continue to be a power house of discovery for future work in mammals.

One of the most fascinating observations in biology is the production of youthful offspring by aging parents. The mechanisms by which this phenotypic rejuvenation can occur are being investigated in the single-celled, budding yeast *Saccharomyces cerevisiae*. This organism has an asymmetrical mitotic division, in which the larger mother cell shows progressive deterioration in the capacity to bud while the smaller daughter cells are born with full replicative potential. At least part of this asymmetry is attributable to a mechanism of spatial quality control (SQC), in which accumulated extrachromosomal rDNA circles (Sinclair & Guarente, 1997) and oxidatively damaged proteins (Erjavec *et al.*, 2007) are selectively retained in the mother cell, a process that requires both the sirtuin Sir2p, a protein deacetylase, and the protein-aggregate remodelling factor Hsp104p (Erjavec *et al.*, 2007). A recent study (Liu *et al.*, 2010) has investigated mechanisms involved in SQC, using synthetic genetic array analysis, which screens for interactions between individually viable mutations that result in synthetic lethality. Such an interaction implies that the two gene products may affect the same process, in this case SQC. A screen for interactions with a Sir2p null mutation identified actin and the polarisome, a protein complex that is involved in the organization of the actin cytoskeleton and consequently required for polarized cell growth. These interactions required the deacetylase activity of Sir2p, and cells lacking Sir2p showed elevated acetylation of the chaperonin CCT, reduced efficiency of actin folding by CCT and elevated levels of non-native actin. Mutants in polarisome components and the myosin V motor protein resulted in reduced efficiency of segregation of protein aggregates and lowered replicative lifespan. Cytoskeletal functions and polarity are thus crucial for the asymmetrical segregation of damaged molecules. A second recent study (Eldakak *et al.*, 2010) has identified a new class of proteins that are retained in the yeast mother cell and that appear to be limiting for replicative lifespan, the MDR transporter proteins (so named because they mediate multidrug resistance in some cancers). These proteins are found in the plasma membrane and eject a variety of toxins from cells. At budding, the mother cell retains its own pool of MDR proteins, while the daughter cell inherits newly synthesized MDRs. Mild over-expression of several of these MDRs increased replicative lifespan, implying that their number or function may decline during aging and limit lifespan.

One unresolved paradox is why loss of asymmetric segregation of damaging molecules in budding yeast leads to shortened replicative lifespan of the bud. Normally, the daughter cell is generated in a youthful state, presumably because the mother retains damaging agents during division. Following this logic, if asymmetry is lost in the absence of altered rates of damage repair, the daughter would inherit more damage at birth but would also retain less damage during subsequent divisions as a mother cell. The observation that lifespan is reduced in cells losing asymmetry may imply that damage inherited in the daughter is more highly detrimental than that accumulating in the mother, perhaps because mothers are more equipped to deal with damage or because impaired asymmetry also leads to impaired repair. These issues remain to be resolved. It will also be important to establish whether these mechanisms of SQC through retention of damaged molecules in the parent by cytoskeletal and polarity function are important in the germ lines and stem cells of multicellular organisms and, if so, to identify the classes of molecules that are affected.

Dietary restriction (DR) can extend lifespan in numerous organisms and can also ameliorate aging-related loss of function and pathology (Mair & Dillin, 2008; Fontana *et al.*, 2010). Dietary restriction in the laboratory model organisms can be implemented using a variety of methods, and it is clear that the mechanisms of lifespan extension can vary with the method used. Although altered nutrition *per se* presumably mediates the extension of lifespan in some cases, it is also becoming apparent that chemosensation, without any change in nutrient intake, can play an important role. For instance, fruit flies subjected to DR show increased lifespan, but if they are exposed to odours derived from live yeast, a standard food source for *Drosophila* in the laboratory and in nature, then some of the extension of lifespan by DR is lost (Libert *et al.*, 2007). Poon *et al.* (2010) now show that sensation of carbon dioxide plays a key role in the response to live yeast. Flies sense the gas using a specific subpopulation of neurons that express two gustatory receptor genes. A null mutation in one of these, *Gr63a*, abolishes the electrophysiological response of the neurons to carbon dioxide, and it also extended the lifespan of female, but not male, flies by 30%. Lifespan was extended mainly by reducing baseline mortality rate rather than reducing the rate of increase with age, similar to the effects of DR in *Drosophila*. The extension of lifespan was lost if *Gr63a* was re-expressed in the neurons using a heterologous expression system, and targeted ablation of the carbon-dioxide-sensing neurons also resulted in an increase in lifespan, demonstrating robust causality. Whereas exposure to odorants from live yeast generally shortened the lifespan of female (but not male) control flies, the mutant *Gr63a* flies did not respond, although flies mutant for a different olfactory mutant, *Orb83b*, which are generally anosmic but retain the ability to sense carbon dioxide, responded normally. Interestingly, *Gr63a* null flies themselves responded normally to DR imposed by dilution of a diet containing killed yeast and sucrose (Poon *et al.*, 2010), a standard method of DR in *Drosophila* in many laboratories, which robustly increases lifespan in multiple strains of flies (Bass *et al.*, 2007; Grandison *et al.*, 2009; Wong *et al.*, 2009; Piper *et al.*, 2010). These results imply that chemosensation of the carbon dioxide from fermentation by live yeast plays an important role in the response of lifespan to this food source, but that other systems, possibly also involving chemosensation, mediate the response of lifespan to killed yeast and/or sucrose. Chemosensation of food can also shorten lifespan in worms subjected to DR (Smith *et al.*, 2008), and it would be interesting to know if olfactory or gustatory mutant mice have longer lifespans or if their lifespans respond differently to DR.

The insulin/Igf/TOR signalling network plays an evolutionarily conserved role in the determination of lifespan in budding yeast, the nematode worm *Caenorhabditis elegans*, the fruit fly *Drosophila melanogaster*, the mouse and, possibly, humans (Fontana *et al.*, 2010; Kapahi *et al.*, 2010; Kenyon, 2010). In the worm, extension of lifespan by reduced activity of the upstream pathway requires the forkhead transcription factor *daf-*16 (Kenyon *et al.*, 1993). Much interest has therefore centred on the roles of orthologous forkhead box O transcription factors in other organisms, including humans (Greer & Brunet, 2008; Partridge & Bruning, 2008; Greer *et al.*, 2009), and in the mechanisms by which altered activity of *daf-16* increases lifespan in the worm. It now transpires that expression of two different, tissue-specific splice variants of *daf-16* are required for the full extension of lifespan (Kwon *et al.*, 2010). The worm has three *daf-16* isoforms, with two (a and b) discovered some time ago and a third (d/f) reported more recently. Some evidence suggested that one of these isoforms, *daf-16a*, was particularly important for the increased longevity of insulin pathway mutants, which is lost in a *daf-16* null background. However, experiments where *daf-16a* alone was re-introduced to worms double mutant for the insulin receptor *daf-2* and for *daf-16* resulted in incomplete rescue of the increased lifespan, leading Kwon *et al.* to take a closer look at the situation. New isoform-specific constructs for RNA interference were used to show that the new *daf-16* isoform, *daf-16d/f*, as well as *daf-16a*, was involved in the extension of lifespan by a mutant insulin receptor. This conclusion was further supported by rescue experiments for the extended lifespan of an insulin receptor mutant in a *daf-16* null background, where both *daf-16a* and *daf-16d/f* were required for full restoration of the lifespan extension, with *daf-16d/f* playing the preponderant role. These two *daf-16* isoforms have different patterns of tissue specificity, with *daf-16d/f* particularly enriched in the intestine, a tissue already known to be important for the action of *daf-16* in the extension of lifespan by reduced insulin signalling (Libina *et al.*, 2003). Rescue experiments with the promoter regions of the daf-16 isoforms swapped indicated that the promoter region, and hence the different patterns of tissue-specific expression, mediated the differing effects on lifespan. The two isoforms also responded differently to altered activity of the two upstream AKT kinases, with daf-16d/f more strongly regulated by AKT-1. The differential inputs and tissue-specific expression of these *daf-16* isoforms may explain the highly pleiotropic effects of this pathway, by allowing specific and spatially differentiated responses to different inputs to insulin signalling, such as nutrition and stress. Worms and flies have only a single gene encoding a forkhead box 0 transcription factor, while mammals have several, which may show similar patterns of differentiation in function to the *daf-16* isoforms.

Aging is accompanied by the accumulation of damage to molecules, cells, tissues and the whole system. At least some of this damage is thought to be causal for loss of function during aging, although an intriguing alternative perspective has been suggested, based upon deleterious effects of TOR activity later in life (Blagosklonny, 2010). Oxygen free radicals (Harman, 1956) have occupied central stage in discussions of damage-induced aging, although the oxidative damage theory has been undermined by recent evidence, as described in last year's Hot Topics (Partridge, 2009). Two studies with *Drosophila* have taken a novel approach, with interesting, but somewhat conflicting, results. Complex 1 of the mitochondrial electron transfer chain, NADH-ubiquinone oxidoreductase, is strongly implicated in the production of superoxide in mitochondria isolated from *Drosophila* and rodents. Direct manipulation of the activities of this complex is hampered by the fact that it consists of over 40 subunits that are encoded by both the nuclear and the mitochondrial genomes. However, the yeast NADH-ubiquinone oxidoreductase Ndi1 is composed of a single, nuclear-encoded polypeptide. Two studies (Bahadorani *et al.*, 2010; Sanz *et al.*, 2010) have examined the effects of introducing this yeast enzyme into *Drosophila* mitochondria *in vivo*. When ubiquitously expressed, enzyme activity, ATP levels and NAD^+^/NADH ratio were all increased. However, one study (Sanz *et al.*, 2010) found reduced superoxide production from isolated mitochondria while the other (Bahadorani *et al.*, 2010) found no change, possibly because different respiratory substrates and methods of measurement were used in the two studies. Ubiquitous expression extended lifespan in one study (Sanz *et al.*, 2010), but only neuronal expression did so in the second (Bahadorani *et al.*, 2010). These somewhat inconsistent results could have been a consequence of differences in expression level, and there are now fly stocks that allow standardization of insertion site and expression level of transgenes (Markstein *et al.*, 2008). This approach to manipulating mitochondrial activity is a promising one for understanding effects of respiratory activity and oxygen free radicals on aging.

Although molecular damage is a marked feature of the aging process, damage at higher levels of organization may be equally important in causing aging. A fascinating new study (D'Angelo *et al.*, 2009) has revealed a new type of damage that occurs at the cellular level and that could play a key role in many postmitotic tissues. Nuclear pores are formed of complexes of nucleoporin proteins, which in dividing cells disassemble at mitosis and reassemble into the newly forming nuclei. D'Angelo *et al.* showed in *C. elegans* that expression of the genes encoding a subset of scaffold nucleoporins is confined to dividing cells, with scaffold proteins produced in the embryo showing life-long stability in the postmitotic cells of the adult worm. RNA interference did not reduce the level of these scaffold nucleoporins in adult somatic cells, nor did it reduce lifespan, even of long-lived insulin receptor mutant worms. A similar down-regulation of expression of scaffold nucleoporins at exit from the cell cycle, and long-term stability of the proteins, was found in mouse cells. Furthermore, these scaffold proteins showed very low turnover compared with other components of the nuclear pore and of the nuclear envelope, such as lamins. D'Angleo *et al.* therefore assessed nuclear pore function during aging, by measuring permeability to fluorescently labelled dextrans of a molecular weight that would normally be excluded. Nuclei isolated from old, but not young, worms and rat brains allowed passage of these molecules, which was prevented by chemical blocking of the nuclear pores. Nuclei from rat cells that failed to exclude dextrans also showed leakage into the nucleus of normally strictly cytoplasmic proteins such as tubulin, which aggregated into large filamentous structures that caused severe chromatin aberrations, and one of the scaffold nucleoporins, Nup93, was lost from these nuclei. It would be interesting to know whether interventions that extend lifespan, such as DR and reduced activity of the insulin/Igf/TOR network, tend to protect scaffold nucleoporins and nuclear pore function during aging.

It is also noteworthy that mutations in *LMNA* cause Hutchinson–Gilford progeria syndrome (HGPS), a severe progeroid disorder characterized by pathologies resembling premature aging (Burtner & Kennedy, 2010). While it is unclear that the molecular causes of HGPS overlap with those of normal aging, it has been reported that A-type nuclear lamins, encoded by *LMNA* and juxtaposed to the nuclear envelope, may regulate nuclear pore function. Moreover, an altered form of lamin A resembling the HGPS mutant is detectable in normal cells and tissues and may increase with age, while defects in nuclear organization increase with age. More studies need to be conducted to determine whether the *LMNA* mutants linked to progeria affect the function of scaffold nucleoporins.

Another potentially widespread type of damage in the cell nucleus during aging has been found in yeast, through loss of chromatin-associated histones (Feser *et al.*, 2010; McCormick & Kennedy, 2010). Levels of histone proteins including histone H3 decline with age, and chromatin immunoprecipitation showed less H3 associated with specific gene promoters, including promoters that are normally both silent and active in aging yeast cells. Tellingly, over-expression of histones led to increased replicative lifespan. This result could imply that looser chromatin packing, and hence elevated expression of many genes, contributes to aging or that histones become damaged during aging and a higher level of histone proteins allows for more frequent replacement. Loss of chromatin structure and de-repression of gene expression could contribute to aging in multicellular organisms, where transcriptional dysregulation during aging has been reported. However, recent findings in the worm and the fly appear at first sight mutually contradictory. A recent report on *C. elegans* (Greer *et al.*, 2010) showed that reduced H3K4 trimethylation, which is usually characteristic of inactive chromatin, increased lifespan of the worm. However, another recent study, in *Drosophila*, showed extended lifespan by reduced chromatin silencing, through lowered trimethylation of lysine 27 in histone H3 by the Polycomb Repressive Complex 2 (Siebold *et al.*, 2010). These results could imply that the effects of chromatin silencing on lifespan are opposite in worm and fly. However, the fly study used heterozygous mutations, which can be a problem in *Drosophila* because of the possibility of heterosis. Nonetheless, it will be important to understand the exact role of chromatin silencing in aging in these two invertebrates.

Aging is the major risk factor for the predominant killer diseases in humans. Animal models of increased lifespan often show evidence of improvement in function and reduction in impact of aging-related diseases, implying that slowing aging also ameliorates the diseases of aging. However, the correlation between increased lifespan and specific improvements in function is not perfect (Partridge, 2010), and it is clear that some models of increased lifespan are not associated with universally improved function during aging (Bhandari *et al.*, 2007) and can even have negative side effects, such as reduced resistance to infection (Clinthorne *et al.*, 2010). It is therefore important to understand the mechanisms linking improved function during aging, resistance to aging-related disease and lifespan. Two recent studies in *C. elegans* are illuminating. In the first, Kauffman *et al.* (2010) developed useful new paradigms to assess associative learning and memory in the worm. During aging, learning ability and long-term memory declined. Reduced insulin signalling and DR both alleviated cognitive decline, but in subtly different ways. A mutation in the insulin receptor *daf-2* improved memory in young adults and maintained the ability to learn during aging, but did not extend long-term memory with age. In contrast, *eat-2* mutants, which reduce pharyngeal pumping and are a model of DR in *C. elegans*, impaired long-term memory in young adults but maintained the memory for longer into older age. The *C. elegans* homologue of the mammalian transcription factor CREB was required for long-term memory. CREB expression declined with age and was higher in young insulin receptor mutant worms and lower in young eat-2 worms and was maintained for longer with age in *eat-2* mutants. The differing effects of these two mutants on long-term memory thus corresponded with their differing effects on expression of CREB. These findings point to the value of understanding the whole signalling network, to identify the modulations that are likely to provide the maximum benefit to specific aspects of aging-related decline.

Also searching for valid generalizations about aging as a risk factor for disease, David *et al.* (2010) investigated protein aggregation as part of the normal aging process in *C. elegans*. Aggregated proteins are present in many neurodegenerative and amyloid diseases, but their possible role in the normal aging process has barely been investigated. David *et al.* found that a significant fraction of the proteins in young worms were insoluble in a strong detergent buffer, and that this fraction increased over threefold with age. Some proteins showed no change in insolubility with age and over-represented among these were cytoskeletal proteins. A subset of 461 proteins showed evidence of a repeatable increase in insolubility with age, which was not associated with an increase in total levels of individual proteins. Rather, those aggregation-prone proteins that were present became more likely to be aggregated as the worms aged. These proteins were enriched for β-sheets, already strongly implicated in promoting protein aggregation, and for involvement in developmental processes, particularly proteostasis itself, including components of the proteasome and chaperones. David *et al.* went on to test the effect on age-related protein aggregation of a mutant in the worm insulin receptor daf-2, already shown to double lifespan and to delay proteotoxicity associated with genetic models of polyglutamine and amyloid beta toxicity in *C. elegans*. Although there was no detectable difference in protein aggregation in young animals, as they aged the *daf-2* mutants showed no increase in protein aggregation with age, and again measurement of the concentrations of specific proteins showed no reduction in total protein present. Thus, in worms, reduced activity of the insulin-signalling pathway may exert a very general beneficial effect on the cellular environment during aging, by reducing the propensity of aggregation-prone proteins to form aggregates. Understanding exactly how this occurs is an interesting future challenge.

These little invertebrates have, for another year, provided many fascinating discoveries for future follow-up in mammals, and they show no sign of losing their ability to provide novel biological insights into mechanisms of aging.
